# Planning is not equivalent to preparing, how Dutch women perceive their pregnancy planning in relation to preconceptional lifestyle behaviour change - a cross-sectional study

**DOI:** 10.1186/s12884-022-04843-4

**Published:** 2022-07-19

**Authors:** Veronique Y. F. Maas, Marjolein Poels, Marleen H. de Kievit, Anniek P. Hartog, Arie Franx, Maria P. H. Koster

**Affiliations:** 1 Department of Obstetrics and Gynaecology, Erasmus MC, University Medical Centre Rotterdam, Doctor Molewaterplein 40, 3015 GD Rotterdam, the Netherlands; 2 Research Agency Care2Research, Niasstraat 7, 1095 TS Amsterdam, the Netherlands

**Keywords:** Preconception Care, Health Beliefs, Lifestyle Behaviours, Pregnancy Planning, Health Promotion

## Abstract

**Background:**

Unhealthy prenatal lifestyle behaviours are associated with adverse pregnancy outcomes, but little is known about what motivates women to comply with preconceptional lifestyle recommendations or consciously plan their pregnancy. Therefore, the objective of this study is to explore the associations between preconceptional lifestyle behaviours, health beliefs and pregnancy planning among Dutch pregnant women.

**Methods:**

In this cross-sectional study based on the data of the APROPOS-II study, 1,077 low-risk pregnant women were eligible for inclusion. Preconception lifestyle behaviours and actively preparing for pregnancy were assessed in relation to planned pregnancies (based on the London Measure of Unplanned Pregnancies) and health beliefs (14 statements). The following preconceptional lifestyle behaviours were assessed through a self-administered questionnaire in the first trimester of pregnancy: fruit intake, vegetable intake, caffeine intake, (second-hand)smoking, alcohol intake, folic acid supplement use and exercise. Data were analysed using multivariate logistic regression analyses.

**Results:**

A total of 921 (85.5%) women in our cohort had a planned pregnancy. However, of these women, 640 (69.5%) adequately used folic acid supplements and 465 (50.5%) women consumed alcohol at any point during pregnancy. Of the women considering themselves ‘healthy enough and not needing preconception care’, 48 (9.1%) women had an adequate vegetable intake, 294 (55.6%) women consumed alcohol at any point during pregnancy and 161 (30.4%) women were either over-or underweight.

**Conclusion:**

Despite consciously planning their pregnancy, most women did not adhere to preconceptional lifestyle behaviour recommendations. Women’s health beliefs and overestimation of their health status seem to interfere with actively planning and preparing for pregnancy. Findings from our study may encourage the development of prospective health-promoting interventions that focus on health beliefs and actively preparing for pregnancy, to improve preconceptional lifestyle behaviours, thereby optimizing the health of future generations.

**Supplementary Information:**

The online version contains supplementary material available at 10.1186/s12884-022-04843-4.

## Background

Currently, it is undeniable that lifestyle behaviours are critical to the health of the public [1]. The prevalence of unhealthy lifestyle behaviours, such as smoking and an unhealthy diet, is a global concern. For instance, in the Netherlands, the percentage of Dutch women who are overweight between the age of 25–45 years, rose from 30% in 2018 to almost 40% in 2018 [2]. A large body of evidence suggests that health behaviours are difficult to change as it includes aspects of habit, automatic responses, conscious choice and calculation [1, 3]. A widely evidenced behaviour change theory by Michie et al. (the COM-B model) proposes that behaviour can be explained by the interaction between capability, opportunity and motivation [4]. A known strong motivator to encourage men and women to change unhealthy lifestyle behaviours is a (future) pregnancy for the benefit of the health of their unborn child [5]. Hence, a pregnancy can be regarded as a window of opportunity for lifestyle interventions [5, 6].

Unhealthy lifestyle behaviours (smoking and alcohol use) during pregnancy are associated with adverse pregnancy outcomes (pregnancy loss, intrauterine growth restriction and low birthweight) as well as adverse long-term neonatal outcomes (impaired cognitive development and behavioural difficulties) [7–9]. Also, there is strong evidence for the health gain of a healthy Body Mass Index (BMI), as a 10% decrease in pre-pregnancy BMI among women who are overweight or women with obesity is associated with at least a 10% lower risk of pre-eclampsia, gestational diabetes, preterm birth, macrosomia, and stillbirth [10]. Since the first two to three months before and after are crucial for optimising gamete function and early placental development, behaviour changes should preferably take place in the periconception period, defined as the 14 weeks before and 10 weeks after conception [7, 11]. However, active preparation for pregnancy is only possible if women also consciously plan their pregnancies.

In Western countries, the rate of planned pregnancies is estimated at around 75–85% of all pregnancies [12, 13]. Characteristics associated with planned pregnancies are parity, educational level, employment, marital status, perceived social support or previous miscarriage [12, 14, 15]. Evidence suggests that women who deliberately plan their pregnancy are more prone to adopt healthier lifestyle behaviours before conception takes place, but on the other hand only a small proportion of women actively prepare for pregnancy (e.g. retrieve health information or visit a healthcare provider) or adhere to the preconceptional lifestyle recommendations e.g. a healthy diet and abstaining from alcohol [16–18]. In a previous systematic review, we identified several barriers for women not to actively prepare for pregnancy nor use preconception care (PCC), for instance, the wish for secrecy, the perceived absence of risks and the social pressure to meet other’s expectations [19].

**Fig. 1:**
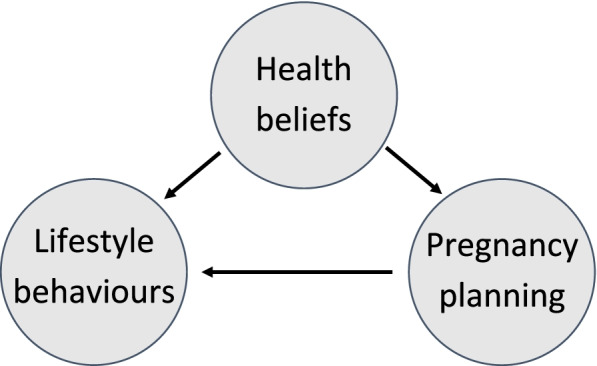
The hypothesized interaction between health beliefs, pregnancy planning and preconceptional lifestyle behaviour

These suggested barriers can be perceived as health beliefs, defined as “what people believe about their health, what they think constitutes their health, what they consider the cause of their illness, and ways to overcome an illness” [20]. While some previous studies hinted towards associations between certain health beliefs and preconceptional behaviour there is still a significant gap in our understanding of which health beliefs drive pregnancy planning or preconceptional lifestyle behaviour change [21–23]. We hypothesize that there is an interchangeable association between health beliefs, pregnancy planning and lifestyle behaviour change (Fig. 1). Insights on how these health beliefs affect pregnancy planning and preconceptional lifestyle behaviours may benefit future health-promoting interventions to improve the preconceptional health of future parents. Therefore, the aim of this was to explore the associations between health beliefs, pregnancy planning and lifestyle behaviour change among Dutch pregnant women.

## Methods

### Study Design and Population

This was a cross-sectional study, based on the data of the APROPOS-II study. A detailed description of the APROPOS-II study, a stepped wedge cluster randomized controlled trial to evaluate the effect of a locally tailored approach for preconception care, has been published elsewhere [24]. For this secondary analysis, only data from the control phase was included to eliminate the possible intervention effect. The APROPOS-II study was approved by the Medical Ethical Review Board (MEC2019-0278) of the Erasmus Medical Centre and written informed consent was obtained from all participants. Participants were pregnant women, recruited in ten independent community midwifery practices (primary care) from six different municipalities in the Netherlands, representing a low-risk population. Dutch women without pre-existing risk factors for adverse pregnancy outcomes receive care at the primary care level by independently practising midwives and are categorized as low-risk [25]. Women with pre-existing risk factors for adverse pregnancy outcomes (e.g. women living with pre-existing hypertension or women who experienced a complicated previous pregnancy or birth) receive care from an obstetrician in a hospital setting and are excluded from participation in this study. All women above the age of 18 years were eligible for inclusion, there were no exclusion criteria to participate in the study. Participants were included at the booking visit by their primary care midwife and were asked to fill out a single questionnaire.

### Data collection

The questionnaire was based on existing validated questionnaires, such as the London Measure of Unplanned Pregnancies (LMUP) and the APROPOS feasibility study [14, 26]. The questionnaire contained 101 questions categorized into four sections: demographics, pregnancy planning, pregnancy preparation, lifestyle behaviours and risk factors and took respondents about 10–15 min to complete. The questionnaire was developed in Dutch, whereafter it was translated into English, Turkish and Polish. One or more of these languages are mastered by the majority of the women in the participating midwifery practices. The English version of the questionnaire is available as Supplemental Material. The questionnaire was distributed between June 2019 until March 2021.

**Fig. 2:**
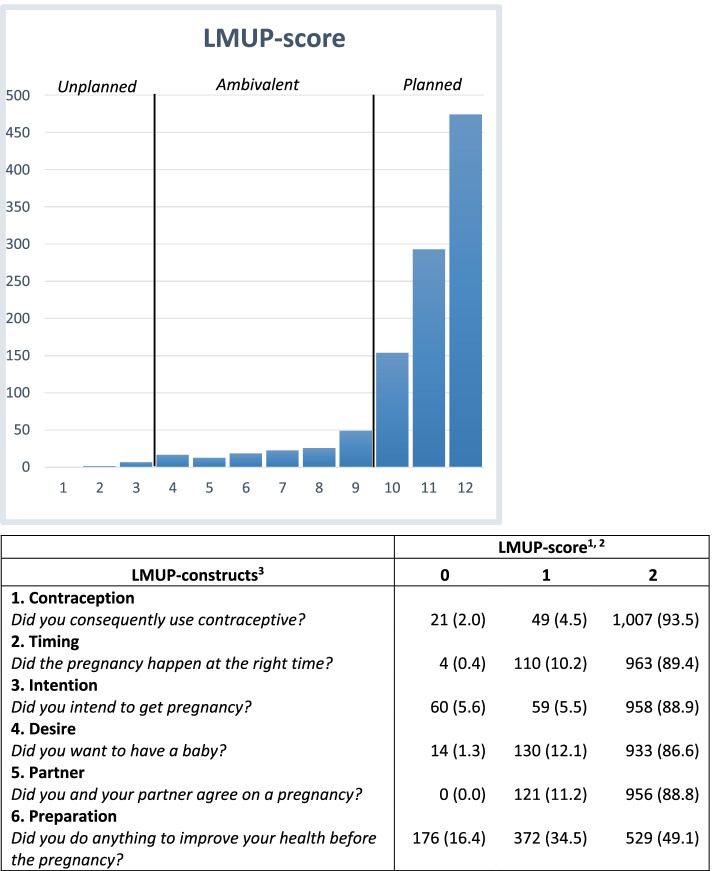
Distribution of LMUP-scores and LMUP-constructs Legend: ^1^Number (%); ^2^A higher score implies a more planned pregnancy; ^3^Short versions of the LMUP-questions, detailed version is available as Supplemental Material

The following characteristics were assessed: age, ethnic background, educational level, pre-pregnancy BMI, parity, gestational age at inclusion, miscarriage in medical history, spontaneous conception and time to conception. In the analysis, ethnic background was categorized as either Dutch or Non-Dutch. Educational level was categorized as highly educated (university or higher vocational education) or medium/low educated (secondary education or lower) based on Dutch classifications [27]. Pre-pregnancy BMI was calculated based on self-reported answers to questions concerning women’s height and weight before conception and was subsequently categorized as women who are underweight (< 18.5 kg/m^2^), a healthy weight range (18.5—24.9 kg/m^2^), women who are overweight (25.0—29.9 kg/m^2^) and women living with obesity (≥ 30.0 kg/m^2^) based on international standards [28]. Gestational age at the time of inclusion was categorized as less than 12 weeks of gestation or 12 weeks of gestation or more. Pregnancy planning was assessed with the official 6-point LMUP-questionnaire, a psychometric measure of pregnancy intention based on lay views and is currently validated in 15 languages [29]. The LMUP-score was calculated based on its official scoring system (Fig. 2), a detailed description of the LMUP-scoring in our study is available as Supplemental Material [14]. A LMUP-score < 4 was categorized as an unplanned pregnancy, a score between 4–9 was classified as ambivalently planned pregnancy and a LMUP-score ≥ 10 was categorized as a planned pregnancy [14].

The following nutrition- and lifestyle behaviour recommendations were assessed based on national guidelines; fruit intake (≥ 2 pieces a day), vegetable intake (≥ 250 g a day), caffeine intake (≤ 1 beverage a day), no smoking, no exposure to second-hand smoking, no alcohol use, folic acid supplements use (400 mg a day for ≥ 4 weeks before conception) and ≥ 150 min per week moderate intensive exercise (e.g. cycling or yoga) or heavy intensive exercise (e.g. running or playing soccer) [30–33]. The initiation of folic acid supplements and the cessation of alcohol and smoking behaviour changes were measured in the periconception period. For folic acid supplement use, the answers were categorized as: started preconceptionally (as recommended), started after pregnancy recognition, or never started. Alcohol use and smoking were also categorized in three categories: no preconceptional use, quit in the preconceptional period, or quit later in the pregnancy/never. Actively preparing for pregnancy is defined by either retrieving PCC-information or visiting a PCC-consultation. Retrieving PCC-information was defined as searching for or receiving any information about a healthy pregnancy before pregnancy. A PCC-consultation was described as being in contact with a healthcare provider about the wish to conceive before an actual pregnancy; in the Netherlands, a general practitioner or midwife is generally appointed to provide a PCC-consultation. Finally, to assess the health beliefs of prospective mothers, the questionnaire contained 14 statements on pregnancy planning and preparing specifically developed for our study. The statements were based on previous Dutch qualitative PCC-studies, such as a systematic review assessing the barriers and facilitators for using PCC [19, 22, 34, 35]. The results of these statements were graded on a 5-Point Likert Scale from 1 (strongly disagree) to 5 (strongly agree). For analysis, the health beliefs were dichotomized as agree (agree or strongly agree) or not-agree (neutral, disagree or strongly disagree).

### Data Analysis

Baseline characteristics of all participants are presented as medians and interquartile range (IQR) for continuous variables or as numbers and percentages for categorical variables. Women answering ‘I don’t know’ to any of the questions were categorized as missing data and excluded from analysis for that specific question. Associations between demographic characteristics and planned pregnancies were analysed using a chi-squared test for categorical variables or by a Mann–Whitney U test for continuous variables. Multivariate logistic regression analysis was performed to identify associations between preconceptional lifestyle behaviour and planning of pregnancy and was adjusted for age, ethnicity, BMI, educational level and parity. A Mann–Whitney U test was performed to assess potential differences in health beliefs between planned and unplanned/ambivalent pregnancies. In addition, differences in the agreement to health beliefs in relation to lifestyle behaviour changes among planned pregnancies were assessed by a chi-squared test or, in case of low frequencies, the Fisher’s Exact test. Finally, to assess what women believe about their health in relation to their actual health status, women’s agreement with the health belief "I believe I am healthy enough myself, so I didn't need any information about becoming pregnant in a healthy way” was compared to their preconceptional lifestyle recommendations. Data were analysed using SPSS version 25.0, *P*-values < 0.05 were considered statistically significant.

### Findings

#### Planned pregnancies

Over a period of 21 months, approximately 7,000 pregnant women were eligible to participate of which 1,158 women actually participated in the study. After the exclusion of 81 incomplete questionnaires for which the LMUP-score could not be calculated, 1,077 women were included in the final analysis. Based on the LMUP-scores, 921 (85.5%) pregnancies were categorized as planned, 147 (13.7%) pregnancies as ambivalent and 9 (0.8%) pregnancies as unplanned (Fig. 2). Almost all women (86.6%—93.5%) scored the maximum of two points on the first five constructs of the LMUP, but 529 (49.1%) women reached the maximum score for adequate preparation of the pregnancy.

The median age of the participants was 31.0 (IQR 29.0–33.0) years, 1,013 (94.7%) women were of Dutch origin, 763 (71.3%) women were highly educated and 564 (52.6%) women were multiparous (Table 1). Demographic characteristics significantly associated with planned pregnancies were: increased age (*p-*value = 0.012), higher education (*p*-value =  < 0.001), parity (*p*-value = 0.046) and a short time to conception (*p*-value =  < 0.001). Table 2 shows that women with planned pregnancies had healthier lifestyle behaviours compared to unplanned or ambivalent pregnancies. Women with planned pregnancies significantly more often used folic acid supplements adequately compared to women with unplanned/ambivalent pregnancies (69.6% versus 12.8%; *p*-value =  < 0.001) and less often consumed alcohol at any point during pregnancy (49.5% versus 37.2%; *p*-value =  < 0.001). In addition, women with planned pregnancies significantly more often retrieved PCC-information (68.7% vs. 21.9%; *p*-value =  < 0.001) and/or visited a PCC-consultation (29.0% vs. 5.8%; *p*-value =  < 0.001) compared to unplanned/ambivalent pregnancies.

**Table 1:** Baseline characteristics

**Demographics**	**Cohort** (*N* = 1,077)	**Unplanned/Ambivalent **(*N* = 156)	**Planned **(*N* = 921)	***P*** **-value**
**Age (years)**^**a**^	31.0 (29.0–33.0)	30.0 (27.0–33.0)	31.0 (29.0–33.0)	**0.012**
Women under 20 years old	5 (0.5)	4 (2.6)	1 (0.1)	
Women between 20–29 years old	362 (33.8)	58 (37.4)	304 (33.2)	
Women between 30–39 years old	686 (64.0)	93 (60.0)	593 (64.7)	
Women above 40 years old	19 (1.7)	0 (0.0)	19 (2.1)	
* Missing data*	*5*	*1*	*4*	
**Ethnic background**				0.063
Women with a Dutch ethical background	1,013 (94.7)	141 (91.6)	872 (95.2)	
Women with a non-Dutch ethical background	57 (5.3)	13 (8.4)	44 (4.8)	
* Missing data*	*7*	*2*	*5*	
**Pre-pregnancy BMI**^**a**^	23.2 (21.3–26.4)	23.5 (21.0–27.3)	23.2 (21.4–26.1)	0.658
Women who are underweight (< 18.5 kg/m^2^)	20 (1.9)	5 (3.2)	15 (1.6)	
Women with a healthy weight range (18.5—24.9 kg/m^2^)	699 (65.5)	88 (57.1)	611 (66.9)	
Women who are overweight (25.0—29.9 kg/m^2^)	218 (20.4)	41 (26.6)	177 (19.4)	
Women with obesity (≥ 30.0 kg/m^2^)	130 (12.2)	20 (13.0)	110 (12.0)	
* Missing*	*10*	*2*	*8*	
**Level of education**				** < 0.001**
Women with a low or moderate level of education	307 (28.7)	64 (41.6)	243 (26.5)	
Women with a high level of education	763 (71.3)	90 (58.4)	673 (73.5)	
* Missing*	*7*	*2*	*5*	
**Parity**				**0.046**
Women who are nullipara	508 (47.4)	62 (40.0)	446 (48.6)	
Women who are multipara	564 (52.6)	93 (60.0)	471 (51.4)	
* Missing*	*5*	*1*	*4*	
**Time to conception**	3.0 (1.0 – 6.0)	1.0 (1.0–3.5)	3.0 (1.0–7.0)	** < 0.001**
< 6 months	722 (70.3)	91 (80.5)	631 (96.0)	
6—11 months	153 (14.9)	12 (10.6)	141 (15.5)	
≥ 12 months	152 (14.8)	10 (8.8)	142 (15.5)	
* Missing*	*50*	*43*	*7*	

^*a*^* Median (interquartile range)*

**Table 2:** Preconceptional lifestyle recommendations among planned and unplanned/ambivalent pregnancies

	**Cohort **(*N* = 1,077)	**Unplanned / Ambivalent** (*N* = 156)	**Planned **(*N* = 921)	*Adjusted P-value*^*a*^
**Nutrition**				
Fruit Intake (≥ 2 pieces)	283 (26.3)	22 (14.1)	261 (28.3)	***0.002***
Vegetable Intake (≥ 250 g)	77 (7.1)	10 (6.4)	67 (7.3)	*0.775*
Caffeine Intake (≤ 1 beverage)	292 (27.1)	37 (23.7)	255 (27.7)	*0.115*
**Lifestyle behaviours**				
Smoking (none)	966 (89.7)	119 (76.3)	847 (92.0)	** < ** ***0.001***
Second-hand smoking (no exposure)	924 (85.8)	114 (73.1)	810 (87.9)	** < ** ***0.001***
Alcohol use (none)	514 (47.7)	58 (37.2)	456 (49.5)	** < ** ***0.001***
Folic acid supplements (≥ 4 weeks before conception)	660 (61.3)	20 (12.8)	640 (69.5)	** < ** ***0.001***
Exercise norm (≥ 150 min per week)	471 (43.7)	59 (37.8)	412 (44.7)	*0.531*
**PCC-behaviours**				
Retrieved PCC-information	655 (60.8)	33 (21.2)	622 (67.5)	** < ** ***0.001***
PCC-consultation	275 (25.5)	9 (5.8)	266 (28.9)	** < ** ***0.001***

Numbers (%), between brackets the preconceptional lifestyle recommendations based on national guidelines

*PCC* Preconception care

^a^Adjusted for; age, ethnicity, BMI, educational level and parity

### Health beliefs

**Fig. 3:**
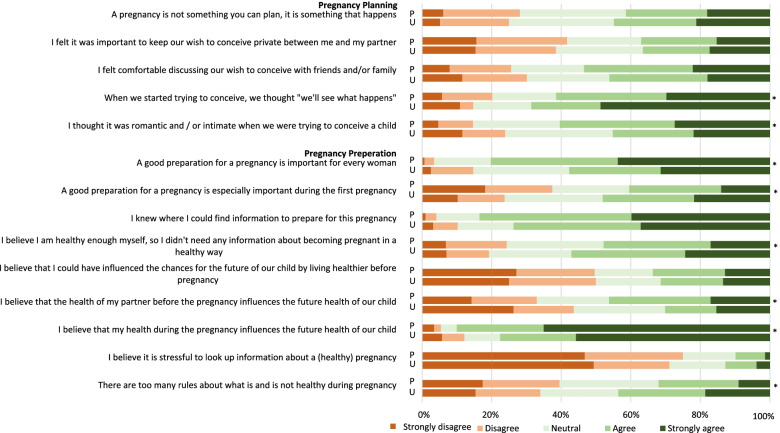
Health beliefs among planned and unplanned/ambivalent pregnancies. Legend: Upper bars represent planned pregnancies (P), lower bars represent unplanned/ambivalent pregnancies (U); *Significant difference by the Mann Whitney U test

Overall, the majority of all women knew where to find PCC-information (82.0%), did not believe it is stressful to retrieve PCC-information (74.5%) and agreed with the statement that ‘good preparation for pregnancy is important for every woman’ (77.0%) (Fig. 3). Women with unplanned or ambivalent pregnancies significantly more often agreed to the health beliefs ‘I believe I am healthy enough myself and don’t need PCC’ and ‘there are too many rules for a healthy pregnancy’ compared to women with planned pregnancies.

Within the planned pregnancy group, women who early initiated folic acid supplements less often agreed to the belief that ‘PCC is especially important for a first pregnancy’ (*n* = 242; 37.8%; *p*-value =  < 0.029) and less often agreed with the belief ‘when we started trying to conceive, we thought "we'll see what happens"’ (*n* = 359; 56.1%; *p*-value =  < 0.001) (Table 3) compared to women who later initiated folic acid supplements or never started. Preconceptional cessation of alcohol use and smoking among planned pregnancies was significantly associated with a low agreement to the ‘when we started trying to conceive, we thought "we'll see what happens" (*n* = 85; 50.0%; *p*-value = 0.001 and *n* = 8; 44.4%; *p*-value = 0.028, respectively). Women who did not quit smoking preconceptionally significantly more often agreed to the belief that ‘there are too many rules about what is healthy in a pregnancy’ (*n* = 33; 45.2%; *p*-value = 0.042).

**Table 3:** Health beliefs stratified by preconceptional lifestyle behaviours among planned pregnancies

	**Folic acid supplement use**				**Alcohol use**				**Smoking**			
	Started PCC ≥ 4 weeks	Started prenatal	Never started	*P-value*	No PCC use	Quit PCC	Quit later/ did not quit	*P-value*	No PCC smoking	Quit PCC	Quit later/ did not quit	*P-value*
	*N* = 640	*N *= 248	*N* = 30		*N* = 286	*N* = 170	*N* = 461		*N* = 829	*N* = 18	*N* = 73	
**Knowledge**												
Visited PCC-consult	217 (33.9)	42 (16.9)	5 (16.7)	** < ** ***0.001***	89 (31.1)	56 (32.9)	119 (25.8)	*0.122*	238 (28.7)	7 (38.9)	20 (27.4)	*0.621*
Retrieved PCC-information	483 (75.5)	122 (49.2)	14 (46.7)	** < ** ***0.001***	194 (67.8)	135 (79.4)	290 (62.9)	** < ** ***0.001***	566 (68.3)	14 (82.4)	41 (56.9)	***0.043***
Knew where to find PCC-information	531 (83.0)	207 (83.5)	27 (90.0)	*0.610*	235 (82.2)	138 (81.2)	391 (85.0)	*0.412*	693 (83.6)	14 (77.8)	30 (82.2)	*0.764*
**Health Beliefs**^a^												
Pregnancy is not something you can plan	259 (40.5)	102 (41.1)	18 (60.0)	*0.105*	127 (44.4)	60 (35.3)	191 (41.4)	*0.161*	337 (40.7)	7 (38.9)	37 (50.7)	*0.243*
Important to keep wish to conceive private	234 (36.6)	94 (37.9)	11 (36.7)	*0.933*	117 (40.9)	64 (37.6)	158 (34.3)	*0.185*	316 (38.1)	4 (22.2)	21 (28.8)	*0.119*
Comfortable discussing wish to conceive	348 (54.4)	129 (52.0)	12 (40.0)	*0.274*	140 (49.0)	94 (55.3)	257 (55.7)	*0.171*	433 (52.2)	13 (72.2)	45 (61.6)	*0.082*
"We'll see what happens"	359 (56.1)	184 (74.2)	21 (70.0)	** < ** ***0.001***	172 (60.1)	85 (50.0)	306 (66.4)	***0.001***	504 (60.8)	8 (44.4)	54 (74.0)	***0.028***
Romantic trying to conceive	387 (60.5)	144 (58.1)	23 (76.7)	*0.143*	164 (57.3)	114 (67.1)	275 (59.7)	*0.112*	511 (61.6)	10 (55.6)	35 (47.9)	*0.066*
PCC important for every woman	539 (84.2)	172 (69.4)	26 (86.7)	** < ** ***0.001***	231 (80.8)	146 (85.9)	359 (77.9)	*0.078*	673 (81.2)	14 (77.8)	51 (69.9)	0.069*
PCC especially important for first pregnancies	242 (37.8)	118 (47.6)	12 (40.0)	***0.029***	116 (40.6)	71 (41.8)	183 (39.7)	*0.892*	333 (40.2)	7 (38.9)	33 (45.2)	*0.695*
Influence of a healthy PCC lifestyle	232 (36.3)	63 (25.4)	14 (46.7)	***0.003***	111 (38.8)	64 (37.6)	135 (29.3)	***0.014***	284 (34.4)	8 (44.4)	18 (24.7)	*0.156*
Influence of partners PCC healthy lifestyle	318 (49.7)	91 (36.7)	16 (53.3)	***0.002***	145 (50.7)	90 (52.9)	188 (40.8)	***0.004***	397 (47.9)	6 (33.3)	22 (30.1)	***0.008***
Influence of a healthy lifestyle in pregnancy	582 (90.9)	221 (89.1)	24 (80.0)	*0.122*	246 (86.0)	153 (90.0)	427 (92.6)	***0.013***	752 (90.7)	15 (83.3)	62 (84.9)	*0.177*
Looking up PCC-information is stressful	62 (9.7)	25 (10.1)	4 (13.3)	0.725*	37 (12.9)	19 (11.2)	35 (7.6)	*0.051*	80 (9.7)	3 (16.7)	8 (11.0)	0.487*
Too many rules for a healthy pregnancy	190 (29.7)	92 (37.1)	12 (40.0)	*0.067*	79 (27.6)	56 (32.9)	158 (34.3)	*0.158*	256 (30.9)	6 (33.3)	33 (45.2)	***0.042***
Healthy enough for a PCC	273 (42.7)	143 (57.7)	23 (76.7)	** < ** ***0.001***	127 (44.4)	75 (44.1)	236 (51.2)	*0.122*	397 (47.9)	8 (44.4)	35 (47.9)	*0.998*

Numbers (%), *PCC* Preconception Care

^a^Agree or strongly agree with this health belief

^*^P-value by the Fishers Exact test

The majority of women with planned pregnancies who did not start folic acid and did not quit alcohol nor smoking preconceptionally agreed to the belief that they are ‘healthy enough and don’t need PCC’ (*n* = 23; 76.7%, *n* = 236; 51.2% and *n* = 35; 47.9%, respectively). However, of all the women in our cohort agreeing to the health belief that they are ‘healthy enough and don’t need PCC’, 48 (9.1%) women adhered to the vegetable intake norm, 294 (55.6%) women consumed alcohol at any point in the pregnancy and 161 (30.4%) women were either over-or underweight (Table 4).

**Table 4:** Preconceptional lifestyle recommendations by the health belief ‘I am healthy enough’

	**"I believe I am healthy enough myself, so I didn't need any information about becoming pregnant in a healthy way"**		
	**Agree**	**Neutral**	**Disagree**
	*N* = 529	*N *= 293	*N *= 254
**Nutrition**			
Fruit Intake (≥ 2 pieces)	151 (28.5)	64 (21.8)	67 (26.4)
Vegetable Intake (≥ 250 g)	48 (9.1)	10 (3.4)	19 (7.5)
Caffeine Intake (≤ 1 beverage)	151 (28.5)	73 (24.9)	67 (26.4)
**Lifestyle behaviours**			
BMI^a^	23.0 (21.2—25.4)	23.5 (21.4—27.8)	23.2 (21.5—27.5)
Women with a healthy weight range (18.5—24.9 kg/m^2^)	368 (69.6)	178 (60.8)	152 (59.8)
Smoking (none)	478 (90.4)	259 (88.4)	228 (89.8)
Second-hand smoking (no exposure)	458 (86.6)	246 (84.0)	219 (86.2)
Alcohol use (none)	235 (44.4)	140 (47.8)	138 (54.3)
Folic acid supplements (≥ 4 weeks before conception)	284 (53.7)	192 (65.5)	183 (72.0)
Exercise norm (≥ 150 min per week)	238 (45.0)	124 (42.3)	108 (42.5)

*Numbers (%)*

^*a*^*Median (interquartile range)*

## Discussion

### Main findings

The results of this study show that up to 85% of women in our cohort had a planned pregnancy, however, the majority of these women did not adhere to preconceptional lifestyle behaviour recommendations. Despite most women reporting stopping their contraceptives in a well-considered manner, discussing a possible pregnancy with their partner and intending, desiring and timing their pregnancy, they do not take action to positively change lifestyle behaviours. We demonstrated some interchangeable associations between preconceptional lifestyle behaviour change, planned pregnancies and health beliefs. We also showed that the pregnant women in our study tended to overestimate their own health status, since most women who agreed with the health belief that they are ‘healthy enough and don’t need PCC’ did not adhere to multiple preconceptional lifestyle recommendations and exhibited many preconceptional risk factors.

### Interpretation

This study confirms that the vast majority of pregnancies are planned and that planned pregnancies are associated with actively preparing for pregnancy [17, 23]. However, as previous studies also showed, only a small proportion of women adhered to the preconceptional recommendations for nutrition and lifestyle behaviour [36, 37]. For example, our results showed that over half of women with planned pregnancies continued to consume alcohol in the preconceptional period, although this has shown to be even higher in previous studies amongst planned in which preconceptional alcohol use ranged from 54.7% to 85.3% [23, 38]. While the evidence on the effect of low-dose preconceptional alcohol consumption on adverse birth outcomes remains contradictory, prenatal alcohol use is a leading, preventable cause of birth defects and developmental disabilities [39–42]. Since 2005, alcohol consumption is discouraged at any stage of (pre)conception and pregnancy by the Health Council of The Netherlands [43]. Previous research also suggests that encouraging women to plan their pregnancy, retrieve PCC-information or visit a PCC-consultation leads to a significant reduction of preconceptional alcohol consumption [16, 42, 44]. While PCC provides a window of opportunity to decrease risk factors, not many couples are aware of the possibility of PCC and, with little regular healthcare engagement before the pregnancy, the uptake of PCC remains low [16, 44–47].

An important finding of our study is that women who agree with the health belief that they are ‘healthy enough and don’t need PCC’ are less likely to plan their pregnancy or change unhealthy preconceptional lifestyle behaviours. This finding promotes the discussion on social norms since women both tend to overestimate how well they are doing themselves, but also how badly others are doing, as observed in a former study identifying the overestimating of alcohol use before and during the pregnancy [48]. Previous studies also found that many women with preconceptional risk factors do not consider themselves as the target population for PCC, while evidence also suggests that almost all couples contemplating pregnancy have at least one preconceptional risk factor [19, 22, 37, 47, 49]. Several studies established three main reasons for this: perceived sufficient knowledge, perceived lack of risk and misunderstanding of the aim of PCC [22, 49]. One study even described how women conducted their own risk analysis and concluded that they were ‘safe’ or that they could handle the risks, while many of these women overestimated their health status [22, 44, 50]. In accordance with the Health Belief Model, future PCC-interventions should incorporate factors such as perception of susceptibility or severity and perceived benefits of lifestyle changes, since they are imperative for changing individual behaviour [51, 52]. For instance, a low perceived threat for developing adverse pregnancy outcomes can be caused by a combination of both a low perceived susceptibility for pregnancy complications (not feeling at risk or overestimating one’s health status) combined with a decreased awareness of the severity of pregnancy complications (unaware of the impact or long-term effects). This low perceived threat to develop adverse pregnancy outcomes could discourage women to change unhealthy preconceptional lifestyle behaviours.

Although the results of our study show that retrieving PCC-information is associated with preconceptional behaviour change, it is known from previous studies that knowledge alone is not enough to change behaviour [1, 18, 21]. Three of the six common errors in prevention policy-making for behaviour change are that behaviour change is (1) neither obvious, (2) nor common sense and (3) that knowledge and information do not drive behaviour [1]. For example, it is not that people are unaware of the importance of a healthy diet, but they experience too many barriers to maintain a healthy diet (e.g. financial constraints, access to supermarkets, personal experiences or social support) [1, 53]. Hence, this emphasizes critical individual differences in human behaviour and decision-making. Therefore, the delivery of health promotion advices as PCC-messages should be tailored to the individual level and match personal health beliefs while simultaneously acknowledging the barriers women face to act upon health-promoting behaviours [54, 55].

Future research should focus on interventions to increase PCC-awareness and -knowledge among the general population and healthcare providers, here lies a specific role for midwives and general practitioners [36, 56, 57]. However, many efforts are needed to achieve healthy behaviour change, since raising awareness and providing PCC-knowledge alone does not remove the experienced barriers to change unhealthy lifestyle behaviours. Health beliefs and lifestyle behaviours of one’s partner should be acknowledged when developing interventions attempting to improve preconceptional lifestyle behaviours. While many PCC-intervention solely focus on improving preconceptional lifestyle behaviours, our results show that advancing womens’ health beliefs by increasing PCC-awareness and encouraging women to plan their pregnancy (as shown in Fig. 1) and discuss social norms, may have the potential to improve the preconceptional health of future parents.

### Strengths and limitations

The long inclusion period (21 months), the distribution of the questionnaire within ten midwifery practices in six different municipalities in the Netherlands, and the availability of the questionnaire in four languages, resulted in a substantial cohort of low-risk pregnant women to be included in this study. Another strength of this study is the extensive questionnaire, evaluating not only preconceptional lifestyle behaviours itself, but also when lifestyle behaviour change took place and how these behaviours were influenced by certain health beliefs.

A potential limitation of this study is that preconceptional lifestyle behaviours were assessed through retrospective self-reported questionnaires in the first trimester of pregnancy. This potentially resulted in recall bias or socially desirable answers. On the other hand, the majority of questions were either multiple-choice, dichotomous questions (yes or no) or statements including a Likert scale (totally disagree – totally agree), lowering the potential risk for over-or underreporting [58]. In addition, as many prospective parents tend to keep their wish to conceive secret, almost all preconceptional health studies have a retrospective cohort design. To minimize recall bias, we included pregnant women at the booking visit in comparison with similar studies where inclusion took place later in pregnancy or even in the post-partum period [23, 44]. In addition, the low response rate (≈15%) makes the results of this study less generalizable since it possible resulted in selection bias. This is a common limitation in many preconceptional studies, leading in our cohort to an underrepresentation of women of a non-Dutch origin and women who obtained a lower education. Future studies should evaluate our results in a more heterogeneous study population.

## Conclusions

The findings of this study show that many women plan their pregnancy, but that the majority of these women do not adhere to preconceptional lifestyle recommendations and tend to overestimate their own health status. Since the reduction of preconceptional risk factors by an individual approach is difficult—if not impossible—for unplanned pregnancies, there is a high need for cost-effective and public health interventions to reduce risk behaviours in the general (reproductive) population, for instance by initiating campaigns to reduce binge drinking or folic acid fortification [7]. Although the design of our study does not allow us to determine causal relationships between pregnancy planning, lifestyle behaviours and health beliefs, the results imply that these three constructs do interact as hypothesized. Future studies should focus on the development of interventions aimed not only to improve the uptake of PCC, but also encourage women to actively prepare for pregnancy. Findings from our study may encourage the development of prospective health-promoting interventions to improve preconceptional lifestyle behaviours, thereby optimizing the health of future generations.

## Supplementary Information

**Additional file 1.**

**Additional file 2.** 

## Data Availability

The dataset for the current study is available from the corresponding author upon reasonable request.

## Abbreviations

BMI: Body Mass Index
IQR: Interquartile range
LMUP: London Measure of Unplanned Pregnancies
PCC: Preconception care
SPSS: Statistical Package for the Social Sciences
