# Supplementary material for: Planning is not equivalent to preparing, how Dutch women perceive their pregnancy planning in relation to preconceptional lifestyle behaviour change - a cross-sectional study
Source: BMC Pregnancy Childbirth. 2022 Jul 19;22:577. doi: 10.1186/s12884-022-04843-4 (PMC9295368; doi:10.1186/s12884-022-04843-4)
Supplement: Supplementary file 2 — Additional file 2. [file 12884_2022_4843_MOESM2_ESM.docx]

**Planning is not equivalent to preparing – how women perceive their pregnancy planning in relation to preconceptional lifestyle behaviour change.**

**Veronique Y.F. Maas^a^, Marjolein Poels^a, b^, Marleen H. de Kievit^a^, Anniek P. Hartog^a^, Arie Franx^a^, Maria P.H. Koster^a^**

^a^ Erasmus MC, University Medical Centre Rotterdam, Department of Obstetrics and Gynaecology, Doctor Molewaterplein 40, 3015 GD Rotterdam, the Netherlands

^b^ Research agency Care2Research, Niasstraat 7, 1095 TS Amsterdam, the Netherlands

**Correspondence:**

Veronique Y.F. Maas,

Department of Obstetrics and Gynaecology

Erasmus MC, University Medical Centre Rotterdam

Doctor Molewaterplein 40, 3015 GD Rotterdam, The Netherlands

E-mail: [v.maas@erasmusmc.nl](mailto:v.maas@erasmusmc.nl)

**Supplemental File 2 - LMUP-Scoring in this study**

1. Contraception

**In the month that I became pregnant…**

- 2 points; I/we were not using contraception
- 1 point; I/we were using contraception, but not on every occasion
- 1 point; I/we always used contraception, but knew that the method had failed (i.e. broke, moved, came off, came out, not worked etc.) at least once
- 0 points; I/we always used contraception

1. Timing

**In terms of becoming a mother (first time or again), I feel that my pregnancy happened at the…**

- 2 points; Right time
- 1 point; Ok, but not quite right time
- 0 points; Wrong time

1. Intention

**Just before I became pregnant...**

- 2 points; I intended to get pregnant
- 1 point; My intentions kept changing
- 0 points; I did not intend to get pregnant

1. Desire

**Just before I became pregnant...**

- 2 points; I wanted to have a baby
- 1 point; I had mixed feelings about having a baby
- 0 points; I did not want to have a baby

1. Partner

**Before I became pregnant…**

- 2 points; My partner and I had agreed that we would like me to be pregnant
- 1 point; My partner and I had discussed having children together, but hadn’t agreed for me to get pregnant
- 0 points; We never discussed having children together

1. Preparation

**Before you became pregnant, did you do anything to improve your health in preparation for pregnancy?**

- Took folic acid ≥4 weeks before conception
- Stopped of cut down smoking
- Stopped of cut down drinking alcohol
- Visited a PCC-consult
- Ate more healthily (at least one of these subcategories)
  - - ≥ 2 pieces of fruit a day
    - ≥ 250 grams of vegetables day
    - ≤ 1 beverage of caffeine a day
- 2 points; ≥ 2 actions
- 1 point; 1 action
- 0 points; no action
